# Protein tyrosine phosphatase receptor-ζ1 deletion triggers defective heart morphogenesis in mice and zebrafish

**DOI:** 10.1152/ajpheart.00400.2021

**Published:** 2021-11-12

**Authors:** Stamatiki Katraki-Pavlou, Pinelopi Kastana, Dimitris Bousis, Despoina Ntenekou, Aimilia Varela, Constantinos H. Davos, Sophia Nikou, Eleni Papadaki, Grigorios Tsigkas, Emmanouil Athanasiadis, Gonzalo Herradon, Constantinos M. Mikelis, Dimitris Beis, Evangelia Papadimitriou

**Affiliations:** ^1^Laboratory of Molecular Pharmacology, Department of Pharmacy, School of Health Sciences, University of Patras, Patras, Greece; ^2^Zebrafish Disease Models Laboratory, Center for Clinical, Experimental Surgery and Translational Research, Biomedical Research Foundation Academy of Athens, Athens, Greece; ^3^Cardiovascular Research Laboratory, Biomedical Research Foundation, Academy of Athens, Athens, Greece; ^4^Department of Anatomy–Histology–Embryology, Medical School, University of Patras, Patras, Greece; ^5^Department of Cardiology, Patras University Hospital, Rio, Greece; ^6^Systems Biology Center, Biomedical Research Foundation, Academy of Athens, Athens, Greece; ^7^Department of Pharmaceutical and Health Sciences, Facultad de Farmacia, Universidad San Pablo-CEU, CEU Universities, Madrid, Spain; ^8^Department of Pharmaceutical Sciences, School of Pharmacy, Texas Tech University Health Sciences Center, Amarillo, Texas

**Keywords:** angiogenesis, cardiac morphogenesis, cardiogenesis, tyrosine phosphatase, zebrafish

## Abstract

Protein tyrosine phosphatase receptor-**ζ**1 (PTPRZ1) is a transmembrane tyrosine phosphatase receptor highly expressed in embryonic stem cells. In the present work, gene expression analyses of *Ptprz1*^−/−^ and *Ptprz1*^+/+^ mice endothelial cells and hearts pointed to an unidentified role of PTPRZ1 in heart development through the regulation of heart-specific transcription factor genes. Echocardiography analysis in mice identified that both systolic and diastolic functions are affected in *Ptprz1*^−/−^ compared with *Ptprz1*^+/+^ hearts, based on a dilated left ventricular (LV) cavity, decreased ejection fraction and fraction shortening, and increased angiogenesis in *Ptprz1*^−/−^ hearts, with no signs of cardiac hypertrophy. A zebrafish *ptprz1*^−/−^ knockout was also generated and exhibited misregulated expression of developmental cardiac markers, bradycardia, and defective heart morphogenesis characterized by enlarged ventricles and defected contractility. A selective PTPRZ1 tyrosine phosphatase inhibitor affected zebrafish heart development and function in a way like what is observed in the *ptprz1*^−/−^ zebrafish. The same inhibitor had no effect in the function of the adult zebrafish heart, suggesting that PTPRZ1 is not important for the adult heart function, in line with data from the human cell atlas showing very low to negligible PTPRZ1 expression in the adult human heart. However, in line with the animal models, *Ptprz1* was expressed in many different cell types in the human fetal heart, such as valvar, fibroblast-like, cardiomyocytes, and endothelial cells. Collectively, these data suggest that PTPRZ1 regulates cardiac morphogenesis in a way that subsequently affects heart function and warrant further studies for the involvement of PTPRZ1 in idiopathic congenital cardiac pathologies.

**NEW & NOTEWORTHY** Protein tyrosine phosphatase receptor **ζ**1 (PTPRZ1) is expressed in fetal but not adult heart and seems to affect heart development. In both mouse and zebrafish animal models, loss of PTPRZ1 results in dilated left ventricle cavity, decreased ejection fraction, and fraction shortening, with no signs of cardiac hypertrophy. PTPRZ1 also seems to be involved in atrioventricular canal specification, outflow tract morphogenesis, and heart angiogenesis. These results suggest that PTPRZ1 plays a role in heart development and support the hypothesis that it may be involved in congenital cardiac pathologies.

## INTRODUCTION

Heart development is a dynamic process that through the spatial and temporal coordination of mechanical and molecular factors regulates cell specification and differentiation, pattern formation, and morphogenesis. Defects in cardiac development may impair cardiac function in health or following stress conditions in the adult (1), and therefore, it is important to uncover molecular pathways that may affect cardiogenesis. Through studies in various experimental models, the significance of numerous cardiac-specific transcription factors has been identified and their role in cardiac development and morphogenesis has been established. Such factors include T-box transcription factors, such as Tbx2, Tbx5, Tbx18, and Tbx20, and Gata4, Hand2, and Pitx2, among others (2–4).

Protein tyrosine phosphatase receptor-**ζ**1 (PTPRZ1) belongs to the receptor-type protein-tyrosine phosphatase family and is overexpressed in human-induced pluripotent stem cells and embryonic stem cells (5). It is also overexpressed in the antler stem cell niche affecting antler regeneration (6) and in glioblastoma stem cells supporting glioblastoma growth (7). PTPRZ1 is not expressed in adult mouse or human liver but its overexpression in liver stem cell niches seems to regulate their mobilization and thus affects the intensity of the ductal reaction following liver injury in various liver pathologies (8). It also mediates the effect of its ligand pleiotrophin (PTN) on hematopoietic stem cell self-renewal in vivo (9). However, its potential role in several organs’ development and function, one among which is the heart, has not been investigated.

PTN has been the most widely studied ligand of PTPRZ1 up to date, but there is also evidence suggesting that PTPRZ1 interacts with several other soluble ligands, such as midkine (MK), fibroblast growth factor 2 (FGF2), interleukin 34 (IL-34), and vascular endothelial growth factor A (VEGFA). Binding of these ligands on PTPRZ1 seems to affect the tyrosine phosphatase activity of the receptor and increase tyrosine phosphorylation of numerous downstream signaling molecules, such as c-Src and Fyn kinases, α_ν_β_3_-integrin, protein kinase C, and phosphoinositide 3-kinase, to control cell adhesion and cell migration (revised in Refs. 5, 10).

PTN is highly expressed in fetal and neonatal but not in normal adult hearts (11), stimulates cardiomyocyte proliferation (12), and is upregulated in peri-infarct and infarcted myocardium (13), in myocardial hypertrophy (13, 14), and in human dilated cardiomyopathy (11). MK has been associated with myocardial infarction and heart failure (15) and has been suggested as a novel target for the treatment of cardiac inflammation in dilated cardiomyopathy (16). VEGFA activates cardiomyocytes and induces cardiac morphogenesis and contractility, whereas high VEGFA levels have been correlated with disease severity and unfavorable prognosis in several cardiovascular diseases (17). FGF2 enhances the differentiation and programming of cultured stem cells and fibroblasts, respectively, to cardiac cells (18). Finally, increased IL-34 levels have been correlated with worse prognosis in acute myocardial infarction (19) and chronic heart failure (20). Although PTPRZ1 is a common receptor, it is not the sole receptor for any of these ligands, and its participation in the described or yet unidentified cardiac functions remains unknown.

In the present work, we used two knockouts of the *ptprz1* orthologs in mice and zebrafish to study the role of PTPRZ1 in cardiac development and function.

## MATERIALS AND METHODS

### Mice and Zebrafish Maintenance, Handling, and Ethics

The *Ptprz1*^−/−^ mice (SV129/B6 strain) were produced by Dr. Sheila Harroch, Institut Pasteur, France (21) and were kindly provided to us by Dr. Heather Himburg and Prof. John Chute at the Division of Hematology/Oncology, Broad Stem Cell Research Center, UCLA, Los Angeles, CA. The animals were bred at the Center for Animal Models of Disease at University of Patras, Greece (EL13B1004) in a controlled environment, that is, 12-h:12-h light/dark cycles and food/water consumption ad libitum. Mouse experiments were approved a priori by the Veterinary Administration of the Prefecture of Western Greece (Approval Protocol No. 388223/1166/8-1-2020) according to Directive 2010/63. All subjects in both *Ptprz1*^−/−^ and *Ptprz1^+/+^* groups demonstrated normal oral intake and everyday activities without any gait abnormalities or other gross neurologic deficits, in line with previous reports (21, 22). Mice were humanely euthanized by excessive CO_2_ and head decapitation, in compliance with the *Guide for the Care and Use of Laboratory Animals*.

Zebrafish (*Danio rerio*) experiments were conducted at Biomedical Research Foundation Academy of Athens (BRFAA) institute on approval by BRFAA ethics committee and the Attica Veterinary Department (EL25BIO003/247914). Adult and embryos zebrafish were raised and maintained under standard laboratory conditions at 28°C and 14-h:10-h day/night cycle according to European Directive 2010/63 of the European Parliament for the protection of animals used for scientific purposes and the European Zebrafish Society (EZS) / Federation of European Laboratory Animal Science Associations (FELASA) guidelines. In this study, the following zebrafish lines were used: *1*) wild-type AB strain; *2*) single or double transgenes *Tg(kdrl:GFP)^s843^*, and *Tg(7xTCFXla.Siam:nlsmCherry)^ia5^*, in which endothelial cells express green fluorescent protein and mesenchymal looking endocardial valve cell express red fluorescent protein in AV channels, respectively; and *3*) the in-house generated *ptprz1b*^−/−^*^aa65^* mutant line, carrying the same transgenes.

### Generation of a Zebrafish *ptprz1b^−/−^* Mutant Line

The *ptprz1* gene is duplicated in zebrafish; *ptprz1a* is located on LG25 (https://zfin.org/ZDB-GENE-090406-1) and *ptprz1b* on LG4 (https://zfin.org/ZDB-GENE-050506-100). Generation of *ptprz1a*^−/−^ and *ptprz1b*^−/−^ zebrafish lines was performed based on CRISPR/Cas9 Genome Editing Technique as previously described (23). For *ptprz1a*^−/−^, we generated CRISPR/Cas9 mutant targeting exon3 but did not observe any phenotype and for *ptprz1b*, we targeted exon2. Both oligos were designed using the online tool CHOPCHOP (https://chopchop.cbu.uib.no) (24). gPtprz1b_ex2_1: 5′- TAGGTCAGCGCAAATTCACAG-3′ and gPtprz1b_ex2_2: 5′- AAACCTGTGAATTTGCGCTGA-3′ oligos were annealed and cloned into a T7-driven expression vector (pT7-gRNA vector, Addgene). For heterozygous mutants generation, F0 carriers were crossed with *Tg(kdrl:GFP)^s843^* and *Tg(7xTCFXla.Siam:nlsmCherry)^ia5^* zebrafish and raised. Tail clipping of adult F1 was performed for genotype identification. PCR products were generated with specific primers to amplify the genomic region of the CRISPR target site in exon2. Genome editing efficiency was confirmed by Sanger sequencing. Primers for genotyping are: Forward: 5′- ACCACATAGCATTATGGAGGCT-3′ and Reverse: 5′- TTTTAAGGCTTTTGGGTCTGAG-3′.

### In Vivo High-Speed Microscope Imaging

High-speed videos (300 frames/s) for 10 s, capturing the continuous changes in ventricular shape during the cardiac cycles, were recorded using the ORCA Flash 4.0 LT camera mounted on a Leica DMIRE2 Inverted Microscope (Leica Microsystems) at a ×20 magnification. For assessment of cardiac function, 5 days postfertilization (dpf), embryos were anesthetized in egg-water with 0.04% tricaine methanesulfonate (400 μL/25 mL egg-water), placed on a small microscope slide with cylindrical chambers, and mounted dorsally in 1.2% low-melting agarose. After agarose has solidified, embryos were positioned dorsally and covered with fresh egg-water for 10 min to remove any residual tricaine and restore normal cardiac contraction.

### Quantification of Zebrafish and Mouse Cardiac Function

For function and structure analyses of zebrafish embryos’ hearts, we used the 10-s high frame rate in vivo imaging as an approach analogous to M‐mode echocardiography for cardiac measurements. Heart rate (HR) was determined by counting the number of heartbeats and multiplying by 6 (beats/min) (25). Individual manual counts were verified by the automated calculation program ZebraBeat (26). For specific ventricular element quantification and further cardiac function characterization of *ptprz1b*^−/−^ embryos, we used a two-dimensional image analysis approach (27). With high-speed videos, a frame-by-frame image analysis was carried out and single regions of interest were identified for each embryo. Three single frames from each video were chosen and ventricle’s width (short axis) and length (long axis) were determined at systole (fully contracted ventricle) and diastole (fully dilated ventricle) phases. End-diastole volume (EDV), end-systole volume (ESV), stroke volume (SV), fraction shortening (FS), ejection fraction (EF), and cardiac output (CO) were calculated in triplicate, based on current practice (25).

Echocardiographic analysis was performed in six *Ptprz1*^−/−^ and six *Ptprz1*^+/+^ male mice anesthetized with isoflurane (5% in 1 L/min oxygen for induction and 1% for maintenance of anesthesia). Transthoracic echocardiography was performed by an experienced sonographer in a blinded manner with a high-frequency ultrasound imaging system (Vevo 2100; Visualsonics, Inc., Toronto, ON, Canada) equipped with 18–38 MHz linear-array transducer (MS400). HR, left ventricular end-diastolic (LVEDD) and left ventricular end-systolic diameter (LVESD), left ventricular posterior wall thickness at end diastole (LVPWd) and end systole (LVPWs), left ventricular internal dimension at end diastole (LVIDd) and at end systole (LVIDs), FS [%FS = (LVEDD − LVESD)/LVEDD × 100%], and left ventricular radius to left ventricular posterior wall thickness ratio (*r*/*h*) were calculated (28).

### PTPRZ1 Tyrosine Phosphatase Inhibitor

MY10 is a selective tyrosine phosphatase inhibitor of human PTPRZ1 that binds to the hydrophobic phosphatase region and leads to a change in the configuration of the protein, blocking the participation of the loop in the catalytic cycle. Its interaction with the active center of human PTPRZ1 takes place in the following amino acids (aa): Ile1826, Tyr1896, Trp1899, Pro1900, Val1904, Pro1905, Val1911, Arg1939, Thr1942, Glu1980, Gln1981, and Phe1984 (29) that are conserved in the active center of zebrafish PTPRZ1 (Supplemental Fig. S1; all Supplemental Figures and Tables are available at http://doi.org/10.6084/m9.figshare.16924447). In this study, MY10 was added into the embryo water of zebrafish at 24-h postfertilization (hpf) to a final concentration of 10 μM. No obvious morphological or developmental phenotype was observed. Alternatively, MY10 was injected at one-cell stage embryos (0.28 pmol/egg). As the inhibitor is dissolved in DMSO, in both cases, DMSO solutions were used for the control embryos. At 72 hpf, embryos were imaged with a Nikon SMZ800 stereoscope to measure their HR. At 5 dpf, high-speed videos were recorded using an ORCA Flash 4.0 LT camera to achieve high-resolution heart function quantification.

### Whole Mount Zebrafish Embryo In Situ Hybridization

In situ hybridization (ISH) was performed in whole mount zebrafish embryos at several developmental stages, using the following antisense probes: *ptprz1b* (ZDB-GENE-050506-100), *notch1b* (DB-GENE-990415-183), *klf2a* (ZDB-GENE-011109-1), and *bmp4* (ZDB-GENE-980528-2059) as previously described (25). For the preparation of the *ptprz1b* probe, we amplified and subcloned the *ptprz1b* cDNA using the primers: forward: 5′-CGG CCA AAC CAC ATC TGT TC-3′ and reverse: 5′-CAA TGC TGG CCT CGA AAA GG-3′.

### Whole Mount Zebrafish Embryo Immunofluorescence and Confocal Imaging

Whole mount zebrafish embryo immunofluorescence was carried out based on Hami protocol with some alterations (30). Zebrafish embryos were fixed in 4% paraformaldehyde overnight at 4°C, washed three times with phosphate buffered saline (PBS), pH 7.4 with 0.5% TritonX-100 (PBST), and treated with proteinase K (10 mg/mL) for 1 h. Following 2-h incubation in blocking solution containing 4% bovine serum albumin (BSA) in PBST at room temperature (RT), primary antibody staining was performed overnight at 4°C, using anti-Eln2a (1:200, gift of Prof. Keeley; 31), anti-MF20 (1:20, Zebrafish International Resource Center, Cat. No. ZDB-ATB-081006-1), and CF633-conjugated phalloidin (1:500, Biotium, Cat. No. 00046). Fluorescent secondary antibodies Alexa Fluor 633 (1:300 in PBST, goat anti-rabbit IgG and goat anti-mouse IgG, Invitrogen) were used for detection as appropriate. Embryos were mounted in 70% glycerol/PBS solution. Imaging was performed using LAS AF software on a Leica TCS SP5 upright confocal microscope.

### Adult Zebrafish Heart Extraction and Immunofluorescence Staining

Adult fish were anesthetized in 0.016% tricaine containing 0.1 M potassium chloride to relax cardiac chamber in diastole position. Isolated hearts fixed overnight at 4°C in 4% paraformaldehyde were cryoprotected into 30% sucrose in PBS overnight and embedded in freezing medium (Tissue-Tek OCT) frozen in liquid nitrogen. Sagittal 10-μm cryosections were generated using a Leica CM3050S cryostat (Leica Microsystems). OCT slides were stained overnight at 4°C with anti-Eln2a (1:300) and CF633-conjugated phalloidin (1:500) (32).

### Measurement of Adult Zebrafish Ventricular Size, Wall Thickness, Hematoxylin & Eosin, and Wheat Germ Agglutinin Staining

Immediately after extraction, adult zebrafish hearts were imaged with a Camera ORCA Flash 4.0 LT mount on Nikon SMZ2800 stereoscope. ImageJ software was used to outline the ventricle, calculate the number of pixels per millimeter, and convert ventricular area (in mm^2^). Ventricular area determination was accomplished by dividing the ventricular area (in mm^2^) by the body length (in mm). Body length was manually measured with a millimeter rule, from the tip of fish mouth to the body/caudal fin juncture (25).

Dissected zebrafish hearts were fixed in 10% formalin overnight at 4°C, following by three washes with PBS, paraffin embedding, and sectioning at 10-μm sequential intervals. Hematoxylin & eosin (H&E) staining was carried out based on standard protocols. For assessment of ventricular wall thickness, tissue sections exhibiting the largest ventricular area were selected and wall thickness was quantified using ImageJ. Wall area thickness was calculated by (ventricular perimeter − ventricular perimeter inside the wall)/body length (25). Wheat germ agglutinin (WGA) staining (1:200, Molecular Probes, Cat. No. W11262) was used to stain cardiomyocyte borders, and DAPI (3 μM, Carl Roth, Cat. No. 6335.1) was used for nuclei visualization. Cardiac sections were imaged by Leica TCS SP5 confocal microscope and cell size was quantified by measuring the cell cross-sectional area (CSA; in μm^2^) using ImageJ software. Quantification was performed in 30–50 randomly selected cardiomyocytes in 2–4 different fields from two independent heart sections.

### Mouse Heart Tissue Processing and Histology

Freshly harvested male mouse hearts were fixed with 10% formalin and were paraffin embedded. Thick sections (4 μm) were cut and stained with H&E, or blocked with PBS containing 3% BSA and 10% fetal bovine serum for 1 h at RT, followed by three washes with PBS and incubation with WGA Alexa Fluor 488 conjugate (5 μg/mL in PBS, Invitrogen, Cat. No. W11261) or with *Griffonia (Bandeiraea) simplicifolia* lectin I (GSL I, BSL I), rhodamine (5 μg/mL in PBS, Vector Laboratories, Cat. No. RL-1102), or with FITC-labeled phalloidin (0.5 μg/mL in PBS, Sigma-Aldrich, Cat. No. P5282) for 1 h at RT in the dark. The tissues were counterstained with Draq5 (1:1,000 in PBS for 15 min at 37°C, Biostatus Limited, Leicestershire, UK, Cat. No. DRS1000) to label nuclei and mounted with Mowiol 4-88 (Merck, Cat. No. 81381). H&E images were obtained under a light microscope (Optech Microscope Services, Ltd., Thames, UK), and fluorescent images were acquired using a Leica SP5 confocal microscope. Cardiomyocyte CSA was measured using the ImageJ software, as previously described (14). Αt least 10 randomly selected cardiomyocytes were evaluated from each tissue section, and results from three different animals in each group were averaged. The *Griffonia simplicifolia* lectin-positive endothelial cells per field were quantified in two to four different fields in one to three slides from three different male mice per group using ImageJ, normalized with the tissue area, and expressed as vascularized area in arbitrary units (AU).

### Quantitative Real-Time PCR

#### Zebrafish.

For quantitative mRNA expression analysis, total RNA was isolated from 5 dpf whole mount zebrafish embryos (15–20 embryos per sample) using TRIzol. cDNA synthesis was performed with PrimeScript RT kit (Takara) and the primers listed in Supplemental Table S1. qRT-PCR was carried out in LightCycler96 system (Roche Life Science) using the KAPA SYBR FAST qPCR kit (KAPA Biosystems) using the following conditions: preincubation for 2 min at 50°C and 10 min at 95°C, two-step amplification for 15 s at 95°C and 1 min at 60°C, for 40 cycles and three melting steps consisting of 60 s at 95°C, 60 s at 65°C, and 10 s at 95°C. All RT-qPCR data are normalized to actin and converted to linear data by the 2^ΔCT^ method. Graphs represent values normalized to control.

#### Mice.

*Ptprz1^+/+^* and *Ptprz*^−/−^ mouse hearts were homogenized, and total RNA was isolated using the Macherey–Nagel RNA isolation kit. cDNA was then reverse transcribed from 100 ng total RNA using the PrimeScript RT Reagent kit (Takara Biotechnology Co., Ltd.). qPCR was subsequently performed using a SYBR Green Real-Time PCR Master mix (Applied Biosystems; Thermo Fisher Scientific, Inc.) on a 7500 Fast Real-Time PCR system (Applied Biosystems; Thermo Fisher Scientific, Inc.). The oligonucleotides used as PCR primers are listed in Supplemental Table S1. The following thermocycling conditions were used for the qPCR: initial denaturation for 5 min at 95°C; followed by 40 cycles of 95°C for 30 s, annealing at 55.5°C for 30 s, extension at 72°C for 40 s; and a final extension step at 74°C for 5 min. Expression levels were quantified using the 2^−ΔΔCq^ method and analyzed using 7500 FAST software (version 2.3; Applied Biosystems; Thermo Fisher Scientific, Inc.) with the expression levels of each mRNA normalized to the endogenous GAPDH mRNA.

### Statistical Analysis

Statistical analysis was carried out using Student’s unpaired *t* test, Mann–Whitney test, and Student’s paired *t* test, as appropriate. More details are included in figure legends.

## RESULTS

### Transcriptome Changes in *Ptprz1^−/−^* versus *Ptprz1^+/+^* Hearts Suggest a Potential Regulation of Heart Development

We have previously shown that RNA sequencing analysis using total RNA derived from *Ptprz1*^−/−^ and *Ptprz1^+/+^* lung microvascular endothelial cells resulted in the identification of 26 transcripts that are significantly changed (22). Gene Ontology (GO) analysis of these data point to a potentially significant effect on heart morphogenesis (Supplemental Fig. S2*A*), due to significant changes in the expression of heart-selective *Tbx2*, *Pitx2*, *Tbx20*, and *Hand2* genes in *Ptprz1*^−/−^ compared with *Ptprz1^+/+^* endothelial cells (Supplemental Fig. S2*B*). To verify differential expression of these genes in the hearts of *Ptprz1*^−/−^ and *Ptprz1^+/+^* mice, we isolated total heart RNA and performed qRT-PCR for these transcripts. As shown in Supplemental Fig. S2*C*, *Tbx2* and *Tbx20* mRNA levels are significantly increased (12- and 4-fold, respectively) and *Hand2* mRNA levels are significantly decreased (∼6-fold) in *Ptprz1*^−/−^ hearts, whereas expression of *Pitx2* does not change.

### *Ptprz1^−/−^* Mice Show Mild but Significant Changes in Heart Function and Enhanced Angiogenesis in the Heart

**Figure 1.**
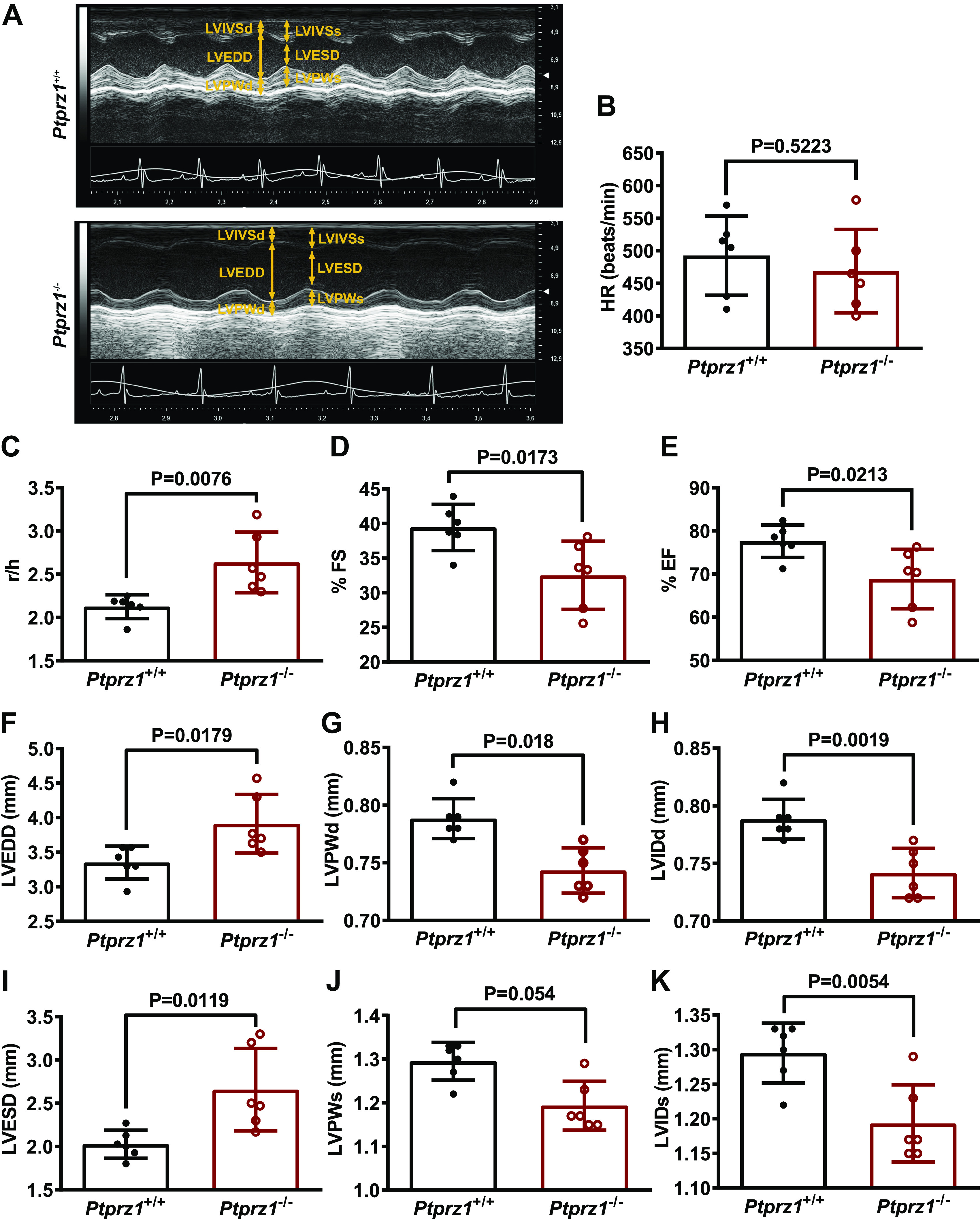
Cardiac ultrasound in *Ptprz1*^−/−^ and *Ptprz1*^+/+^ mouse hearts. *A*: representative cardiac ultrasound images of the LV at the level of the papillary muscle before the mitral valve. These are short axis mode images, which show from above to below the interventricular wall, the cavity, and the posterior wall of the left ventricle. *B*–*K*: quantitative data derived from such images are shown and data in all cases are expressed as means ± SD from 6 *Ptprz1*^−/−^ and 6 *Ptprz1^+/+^* mice at the age of 3 mo. Statistical analysis was performed by Student’s unpaired *t* test. HR, heart rate; LV, left ventricle; LVEDD, left ventricular end-diastolic diameter; LVESD, left ventricular end-systolic diameter; LVIDd, left ventricular internal dimension at end-diastole; LVISd, left ventricular internal dimension at end systole; LVPWd, left ventricular posterior wall thickness at end-diastole; LVPWs, left ventricular posterior wall thickness at end systole; %EF, percent ejection fraction; %FS, percent fractional shortening; *r*/*h*, left ventricular radius to left ventricular posterior wall thickness ratio.

Echocardiography analysis was performed on six male *Ptprz1*^−/−^ and six male *Ptprz1^+/+^* mice at the age of 3 mo, and the data are shown in Fig. 1 and Supplemental Movies S1 and S2 (all Supplemental Movies are available at http://doi.org/10.6084/m9.figshare.15047994). There is no statistically significant difference in the HR or the LV mass between the two genotypes. Both EF and FS are significantly decreased in the *Ptprz1*^−/−^ compared with the *Ptprz1^+/+^* mice, showing a reduced overall LV function of the *Ptprz1*^−/−^ hearts. Both LVEDD and LVESD were significantly increased in *Ptprz1*^−/−^ mice, while the LVPW and the LVID thickness in both end systole and diastole were decreased. Moreover, the r/h index was increased in the dilated hearts of *Ptprz1*^−/−^ compared with *Ptprz1^+/+^* mice.

**Figure 2.**
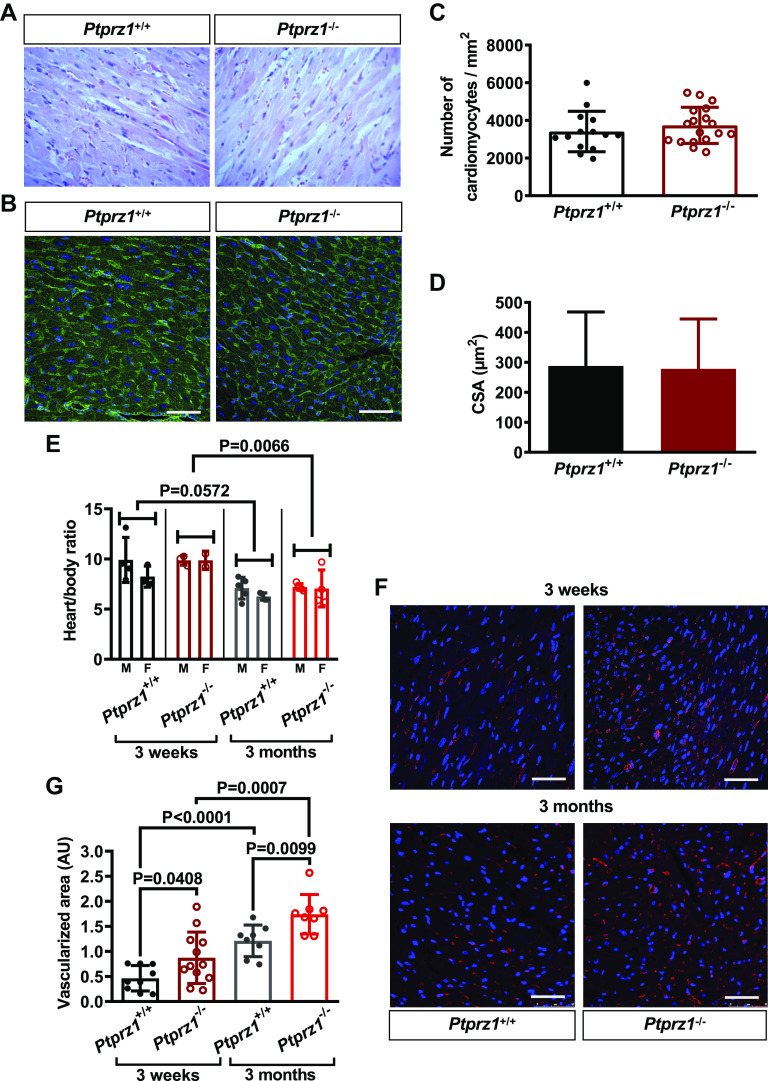
Histological evaluation and angiogenesis in *Ptprz1*^−/−^ and *Ptprz1*^+/+^ mouse hearts. *A*: representative images at ×40 magnification of H&E staining of heart tissue sections from 3 *Ptprz1*^−/−^ and 3 *Ptprz1^+/+^* male mice at the age of 3 mo. *B*: representative images of WGA-stained heart tissue sections from *Ptprz1*^−/−^ and *Ptprz1^+/+^* male mice. Scale bar corresponds to 50 μm. *C* and *D*: the number of cardiomyocytes per mm^2^ and the CSA of 10 cardiomyocytes per slide was measured in at least 4 different slides from each of 3 different mice from each group. Results are expressed as means ± SD of the number of cardiomyocytes per mm^2^ (*C*) or the calculated cardiomyocyte CSA expressed in μm^2^ (*D*). *E*: calculated heart to body ratio expressed as means ± SD (*n* ≥ 5) at 2 different ages is shown. Statistical analysis was performed by Student’s unpaired *t* test. Both male (M) and female (F) mice were used, and no difference related to sex has been identified. *F*: paraffin-embedded hearts from *Ptprz1*^−/−^ and *Ptprz1^+/+^* male mice at the age of 3 wk or 3 mo were stained with rhodamine-conjugated *Griffonia simplicifolia* for endothelial cells (red). Nuclei were stained with Draq5 (blue). Representative pictures are shown, and scale bars correspond to 50 μm. *G*: *Griffonia simplicifolia* lectin-positive endothelial cells per heart area were measured in 2–4 different fields/photo from 3 different animals from each group. Results are expressed as means ± SD of the vascularized cardiac tissue area in arbitrary units (AU). Statistical analysis in all cases was performed by Student’s unpaired *t* test. CSA, cross-sectional area; H&E, hematoxylin & eosin ; WGA, wheat germ agglutinin.

Histological evaluation of the hearts at the same age showed no significant differences in the tissue architecture and no signs of fibrosis (Fig. 2, *A* and *B*). The number of cardiomyocytes or the cardiomyocyte CSA is similar between *Ptprz1*^−/−^ and *Ptprz1^+/+^* mice (Fig. 2, *C* and *D*), and the adjusted heart weight ratio is not significantly different between *Ptprz1*^−/−^ and *Ptprz1^+/+^* mice, although it is significantly decreased at 3 mo versus 3 wk at both genotypes (Fig. 2*E*). Angiogenesis is significantly higher in the *Ptprz1*^−/−^ compared with the *Ptprz1^+/+^* mice hearts at both 3 wk and 3 mo (Fig. 2, *F* and *G*), and the vessel network seems to be disorganized, as also implied by phalloidin staining of heart tissue sections (Supplemental Fig. S3). Angiogenesis is also significantly increased at 3 mo versus 3 wk at both genotypes (Fig. 2*G*).

### Generation of ptprz1b Knockout Zebrafish Line

To investigate the functional role of *ptprz1* in cardiovascular system development, we used CRISPR-Cas9 editing technology. The gene is duplicated in zebrafish and we targeted *ptprz1a* and *ptprz1b* independently, following previously reported guidelines (23). A *ptprz1a* allele was generated (Supplemental Fig. S4) but an in-cross of heterozygous carriers showed no phenotype, so it was not further studied. A similar procedure was followed for *ptprz1b* (Supplemental Fig. S5*A*) and the *ptprz1b* F1 founder that carried a 2-bp deletion (Supplemental Fig. S5, *B* and *C*) was selected for further analyses. Expression of *ptprz1b* in 5 dpf larvae was significantly decreased in *ptprz1b*^−/−^ compared with the *ptprz1b^+/+^* embryos (Supplemental Fig. S5*D*) in F2, suggesting the activation of a nonsense-mediated decay. In silico translation analysis verified that the 2-bp deletion results in a premature stop codon in the Ptprz1b protein. Subsequently, it is predicted that the mutant protein would have 65 aa, sharing only 34 aa with the wild-type protein (Supplemental Fig. S5*E*).

### *Ptprz1b*^−/−^ Larvae Display Significantly Defective HR, Enlarged Ventricles, and Defected Contractility

**Figure 3.**
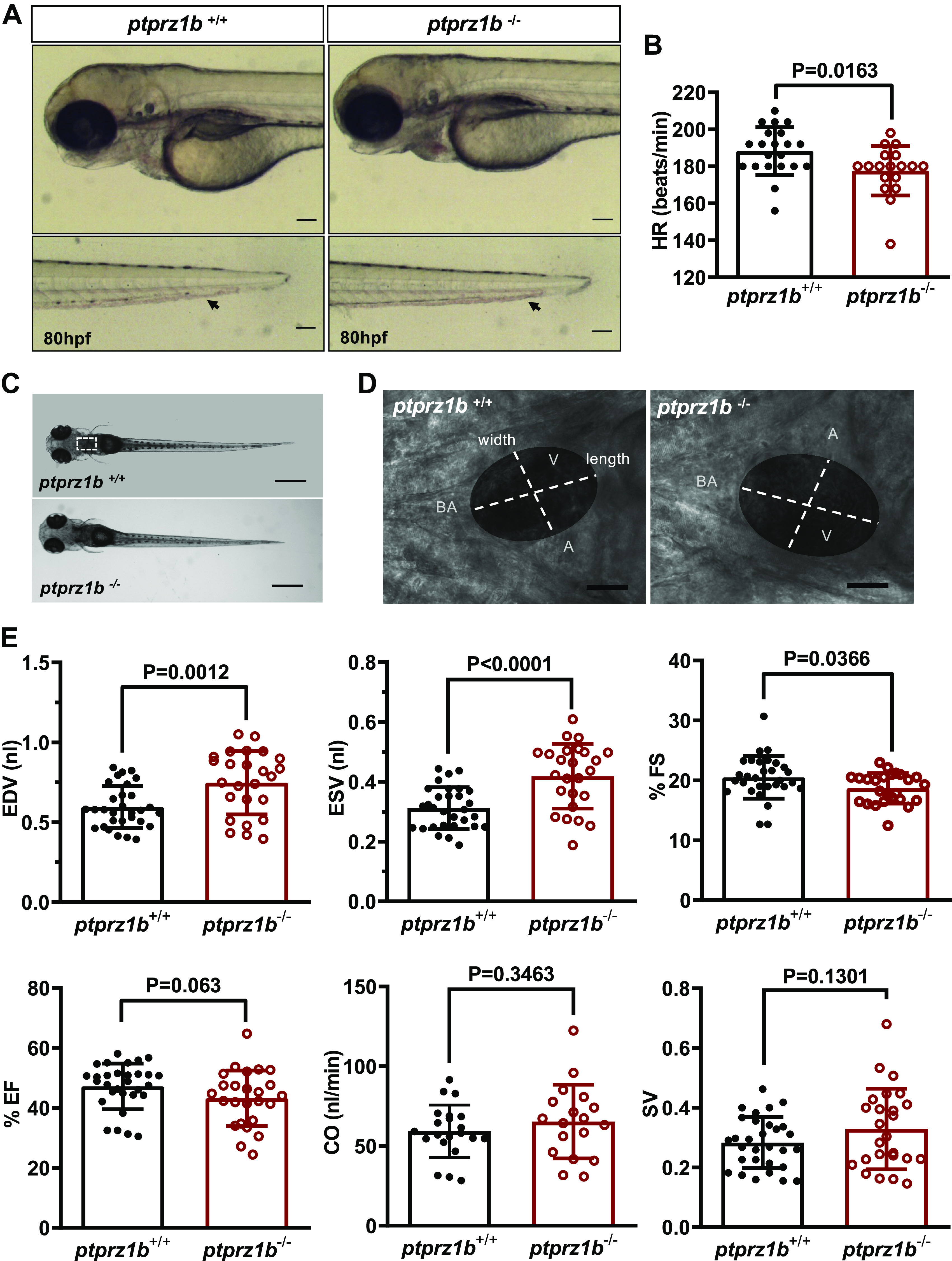
*Ptprz1b*^−/−^ zebrafish larvae have defective heart rate (HR), enlarged ventricles, and defective contractility. *A*: *Ptprz1b^+/+^* and *ptprz1b* ^−/−^ embryos at 80 hpf exhibit similar morphology (images are lateral views, anterior to the *left*, dorsal to the *top*). *Ptprz1b* ^−/−^ embryos exhibit lower blood flow (black arrows, embryo tails). Scale bars correspond to 100 μm. *B*: HR expressed as means ± SD (*ptprz1b^+/+^*: *n* = 30 and *ptprz1b*^−/−^: *n* = 20); statistical analysis was performed by Student’s unpaired *t* test. *C*: microscope images illustrating the morphology of 5 dpf *ptprz1b^+^*^/+^ and *ptprz1b*^−/−^ mutants as seen from a left lateral. The heart area is marked with a white dashed box. Scale bars correspond to 500 μm. *D*: representative images of 5 dpf embryos’ hearts (vertical view) at fully diastole phase. Images are individual frames from high-speed microscope imaging, recording embryonic cardiac beating. The ventricular area of the heart is highlighted, with the length (long axis) and width (short axis) of the ventricle indicated by dashed lines. Scale bars correspond to 50 μm. *E*: quantification of cardiac function at 5 dpf. Results are expressed as means ± SD (*ptprz1b^+/+^*: *n* = 30, *ptprz1b*^−/−^: *n* = 20) and statistical analysis was performed by Student’s unpaired *t* test and Mann–Whitney test. A, atrium; BA, bulbous arteriosus; CO, cardiac output; dpf, days postfertilization; EDV, end-diastole volume; ESV, end-systolic volume; %EF, percent ejection fraction; %FS, percent fractional shortening; SV, stroke volume; V, ventricle.

*Ptprz1b* is expressed during the first embryonic stages in different tissues including the heart, as we show with in situ and RT-PCR analyses (Supplemental Fig. S6). *Ptprz1b*^−/−^ embryos exhibit no obvious phenotype compared with the *ptprz1b^+/+^* embryos, develop normally, and are viable to adulthood (Fig. 3*A*). To investigate the functional viability of maternal zygotic homozygous *ptprz1b*^−/−^, embryos from an F2 *ptprz1b*^−/−^ in-cross were collected and observed. No obvious phenotypic defect was detected in these embryos. However, we detected a decreased HR at 80 hpf (Fig. 3*B*; Supplemental Movies S3 and S4), resulting in a reduced blood circulation throughout mutant embryos’ bodies (Supplemental Movies S5 and S6).

Embryos’ functional and structural analysis was performed using high-speed imaging (Fig. 3*C*). We measured ventricle’s width (short axis) and length (long axis) both at systole (fully contracted ventricle) and diastole (fully dilated ventricle) phases for three individual cardiac cycles (Fig. 3, *D* and *E*; Supplemental Fig. S7*A*). Remarkably, elevated end-diastolic and end-systolic volumes in *ptprz1b*^−/−^ embryos appear to result from enlarged ventricles, with strikingly reduced FS. SV and CO present a slight but not significant increase (Fig. 3*E*). Likewise, 5 dpf embryos were stained with MF20 antibody, which recognizes a sarcomere myosin heavy chain epitope. Measurements of ventricle-MF20-positive area confirmed the dilated ventricle phenotype (Supplemental Fig. S7, *B* and *C*). Finally, Titin gene (*ttn2*) expression is also significantly decreased in *ptprz1b*^−/−^ embryos (Supplemental Fig. S7*D*). Collectively, these findings suggest that *ptprz1b* mutation in zebrafish affects heart development and contractility similarly to its effect in mice.

**Figure 4.**
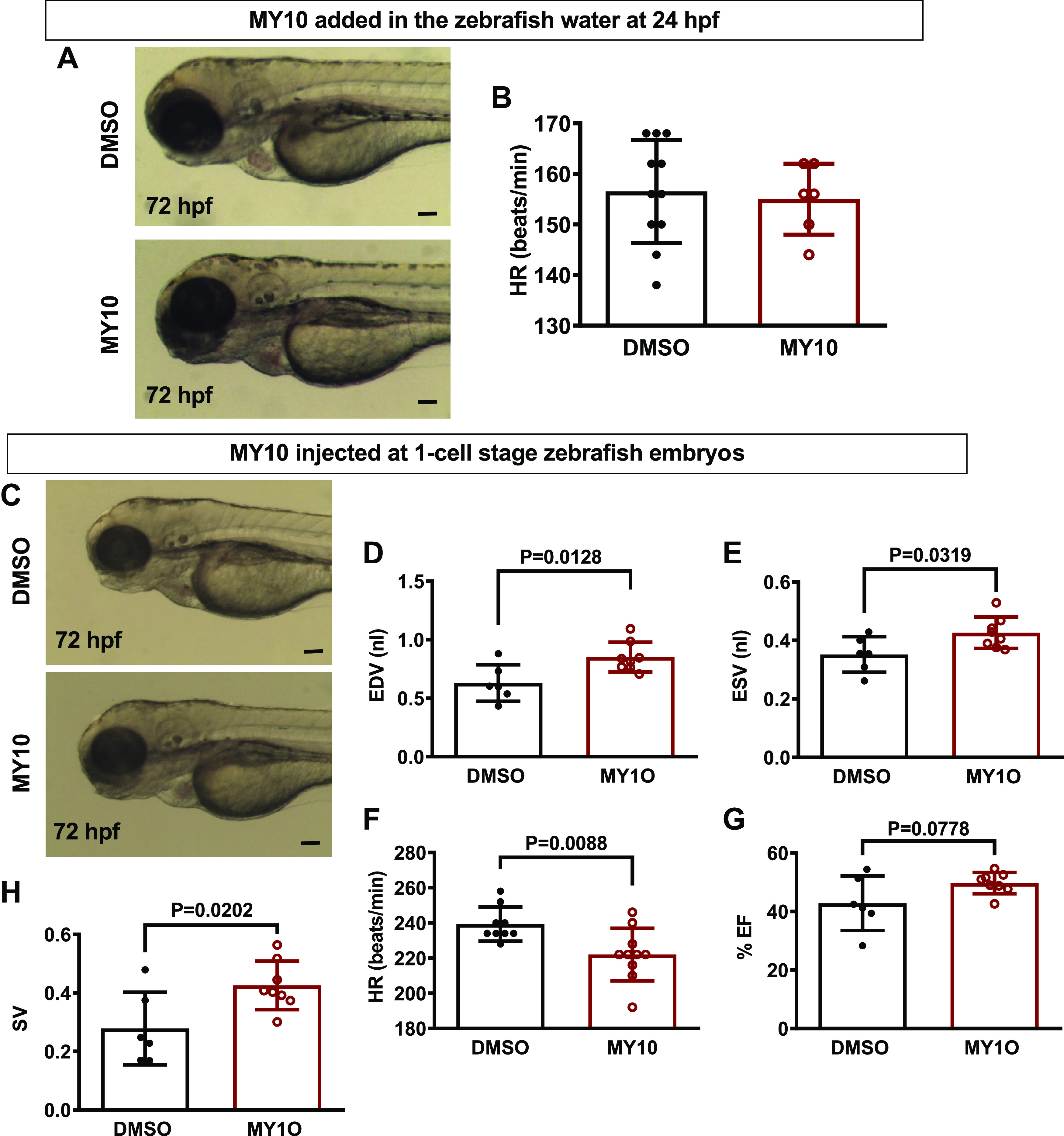
The effect of the protein tyrosine phosphatase receptor-**ζ**1 (PTPRZ1)-selective tyrosine phosphatase inhibitor MY10 in zebrafish heart. *A*: MY10 was added in the water of 24 hpf zebrafish embryos and the latter were observed under a stereoscope and photographed at 72 hpf. Scale bar corresponds to 100 μm. *B*: HR is expressed as means ± SD of heart beats/min (DMSO 0.1%: *n* = 9, MY10 10^−5^ M: *n* = 10). *C*: MY10 was injected at 1-cell stage embryos and the injected embryos were observed and photographed at 72 hpf. Scale bar corresponds to 100 μm. *D*–*H*: quantification of cardiac function of injected embryos at 5 dpf. Data are expressed as means ± SD (DMSO: *n* = 6, MY10: *n* = 8). Statistical analysis was performed by Student’s unpaired *t* test. dpf, days postfertilization; EDV, end-diastole volume; ESV, end-systolic volume; hpf, hours postfertilization; HR, heart rate; %EF, percent ejection fraction; SV, stroke volume.

The addition of the selective PTPRZ1 inhibitor MY10 into embryos egg-water after the formation of the heart tube and the initiation of heart beating at 24 hpf did not affect heart morphogenesis or heart function (Fig. 4, *A* and *B*). However, after one-cell microinjection of MY10, we observed differences in embryo cardiac function like the ones of the mutant line (Fig. 4, *C*–*H*), further pointing out to a developmental role for *Ptprz1b* function. These results confirm the cardiac dilation phenotype of the mutant and attribute the cardiac pathology to the phosphatase activity inhibition during cardiac development.

### *Ptprz1b*^−/−^ Zebrafish Exhibit Reduction and Misregulation of Developmental Cardiac Markers

**Figure 5.**
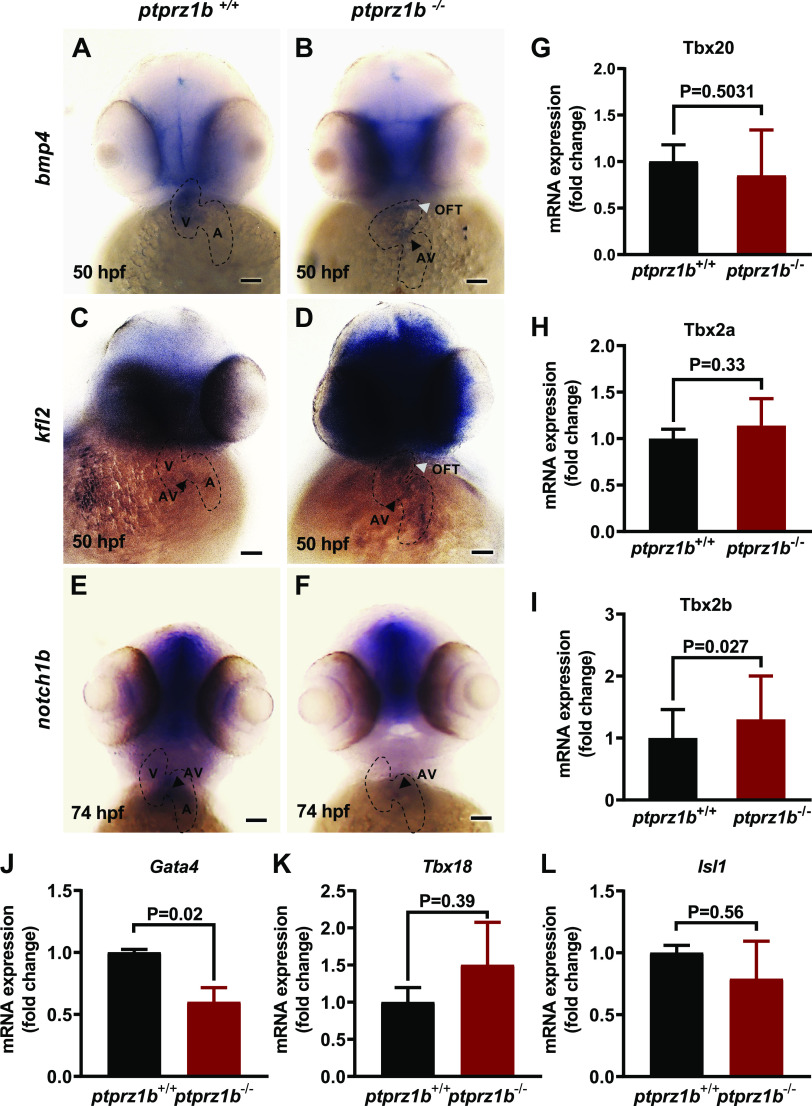
Expression of cardiac markers during zebrafish cardiac development. *A*–*F*: expression of cardiac genes using whole mount antisense ISH. The boundaries of the heart are drawn with dashed lines, black arrowheads indicate the location of the atrioventricular valves (AV), whereas white arrowheads indicate the outflow tract (OFT). Scale bars correspond to 100 μm. *G*–*I*: T-box family genes relative expressions. *J*–*L*: cardiac development regulator genes relative expressions. For *G*–*L*, RNA was isolated from *ptprz1b^+/+^* and *ptprz1b*^−/−^ embryos at 3 and 5 dpf and mRNA levels were determined by qRT-PCR and calculated by the 2^ΔCT^ method. Results are expressed as means ± SD (*n* = 4) and statistical analysis was performed by Student’s unpaired *t* test. hpf, hours postfertilization; ISH, in situ hybridization; OFT, outflow tract; A, atrium; V, ventricle; AV, atrioventricular canal.

To identify developmental cardiac markers that may be affected in *ptprz1b*^−/−^ zebrafish hearts, we performed expression analysis for several such markers. *Bmp4* is a flow-dependent myocardial gene that is expressed in the whole heart tube and gradually, from 48 to 54 hpf, becomes restricted in the atrioventricular canal (AVC). The endocardial markers *klf2a* and *notch1b* are expressed at the anterior part of the heart and are progressively localized in the AVC and the outflow tract (OFT) (32). In *ptprz1b*^−/−^ zebrafish, *bmp4* expression is considerably decreased and mainly localizes in the ventricle borders (Fig. 5, *A* and *B*), whereas *klf2a* is ectopically expressed and does not attain a restricted expression pattern at 50 hpf (Fig. 5, *C* and *D*). At 74 hpf, *notch1b* expression in AVC (Fig. 5, *E* and *F*), as well as throughout the embryo body (Supplemental Fig. S8*A*), is low. We also analyzed the expression levels of T-box family genes, which are crucial regulators of early cardiac morphogenesis. We identified *tbx2b* mRNA levels to be significantly elevated in *ptprz1b*^−/−^ zebrafish at 5 dpf, whereas *tbx2a* and *tbx20* mRNA levels are unaffected (Fig. 5, *G–I*). Likewise, we detected a decrease at mRNA expression levels of the cardiac regulator *gata4* (Fig. 5*J*), whereas no difference was detected at *tbx18* and *isl-1* gene expression levels (Fig. 5, *K* and *L*). The valvular-related defects were confirmed by the decrease of *Tg(7xTCFXla.Siam:nlsmCherry)^ia5^* (TCF)-positive (mesenchymal) AVC cells in *ptprz1b*^−/−^ embryos (Supplemental Fig. S8, *B* and *C*). Taken together, these results suggest that *ptprz1b* is involved in cardiac morphogenesis and AVC specification during early heart development.

### OFT Morphogenesis in *ptprz1b^−/−^* Embryos

**Figure 6.**
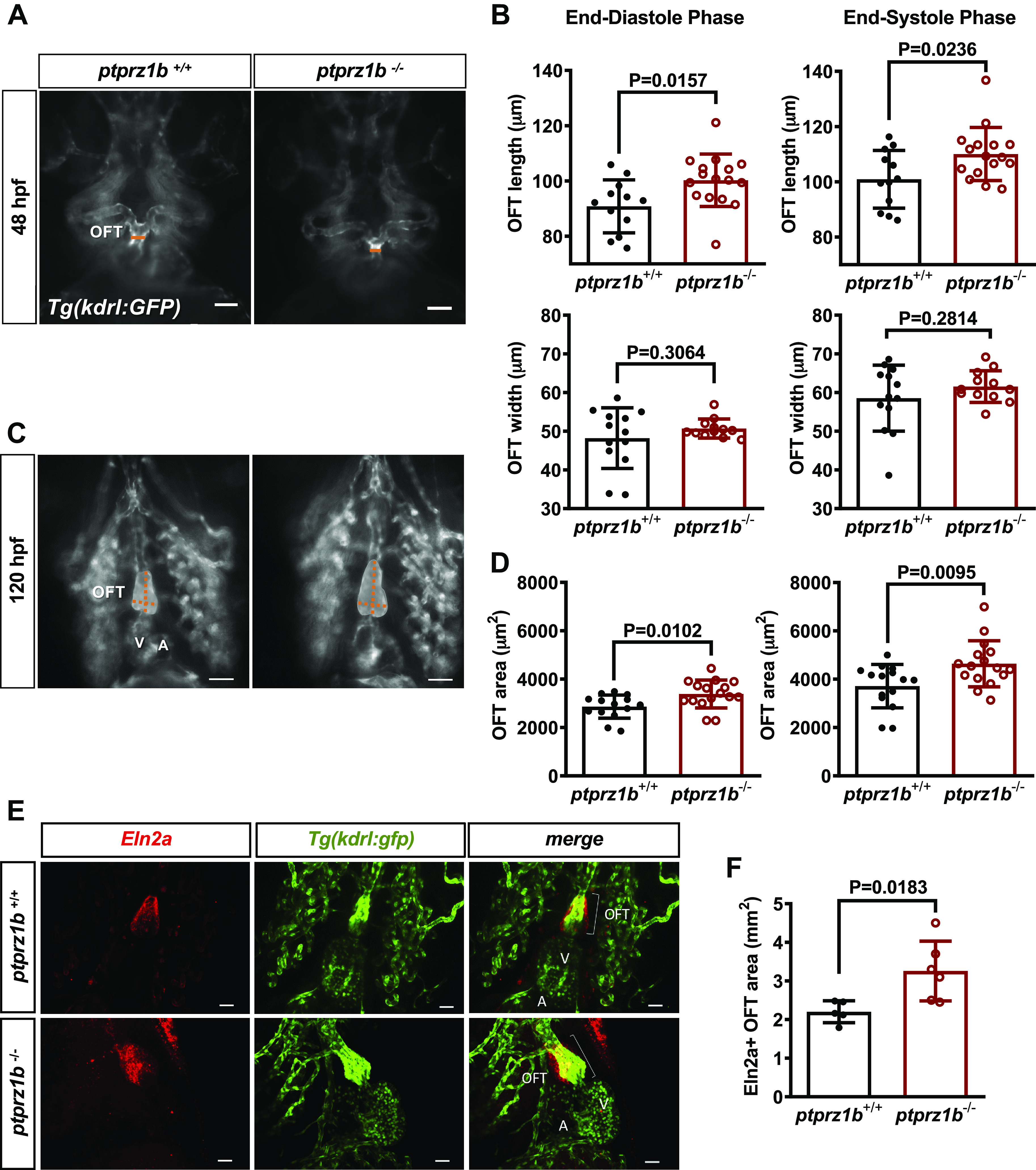
OFT morphogenesis in *ptprz1*^−/−^ zebrafish embryos. Inverted microscope images at 48 (*A*) and 120 hpf (*C*; ventricular orientation) are shown. The width and length of the developing OFT are indicated by the orange lines and the OFT area is displayed in gray. Scale bars correspond to 50 μm. *B*: quantification of the OFT area at 48 hpf, based on length and width measurements at OFT maximum (end-systolic phase) and minimum (end-diastolic phase) dimensions. *D*: quantification of the OFT area at 120 hpf. Data in *B* and *D* are expressed as means ± SD (*n* = 16). Statistical analysis was performed by Student’s unpaired *t* test. *E*: 7 dpf *ptpr1b*^−/−^ and *ptpr1b*^+/+^ embryos exhibit robust Eln2a^+^ expression in the OTF (Eln2a: smooth muscle cells). Scale bars correspond to 25 μm. *F*: quantification of the Eln2a^+^ area expressed as OFT/BA area (mm^2^). Data are expressed as means ± SD (*ptprz1b^+/+^*: *n* = 5, *ptprz1b*^−/−^: *n* = 6) and statistical analysis was performed by Student’s unpaired *t* test. A, atrium; BA, bulbus arteriosus; dpf, days postfertilization; hpf, hours postfertilization; OFT, outflow track; V, ventricle.

To investigate whether *ptprz1b* is involved in OFT development, we exploited the *Tg(kdrl:GFP)^s843^* transgenic zebrafish line and quantified the size of the OFT at 48 and 120 hpf. The OFT of *ptprz1b*^−/−^ embryos was significantly enlarged when compared with *ptprz1b^+/+^* in both cases (Fig. 6, *A*–*D*), due to OFT length elongation (Fig. 6*B*). Normally, the bulbus arteriosus (BA), which is located within the OFT structure, is surrounded by a layer of smooth elastic cells. At 7 dpf, the OFT structure has developed normally, and the smooth Eln2a-positive BA cells are visible in both genotypes (Fig. 6*E*). By measuring the OFT surface, we found that *ptprz1b*^−/−^ embryos have extended BA/Eln2a-positive area (Fig. 6*F*), suggesting involvement of Ptprz1b in the development and morphogenesis of the cardiac OFT.

### Zebrafish *ptprz1b^−/−^* Embryos Show Disorganized Angiogenesis

Τo investigate the effects of *ptprz1b* mutation on early vascular development, we focused on brain blood vessel angiogenesis, which is mainly responsible for brain perfusion in young zebrafish individuals. Normally during early brain development, new vessels grow from the main lateral vessels to the basilar artery, a process that leads to the formation of central arteries (CtAs; Supplemental Fig. S9*A*). At 48 hpf, confocal imaging revealed that *ptprz1b*^−/−^ embryos had reduced number of CtAs vascular sprouts compared with the *ptprz1b^+/+^* embryos (Supplemental Fig. S9, *B* and *C*) but at later developmental stages, the number of CtAs vessels restored, revealing an early delayed angiogenesis. Almost 80% of *ptprz1b*^−/−^ embryos showed a differential mesencephalic artery vessel arrangement, resulting in the formation of an alternative structure with shape-V instead of the normal shape-Y structure, at the upper part of the vascular brain network (Supplemental Fig. S9*D*).

### *Ptprz1b^−/−^* Adult Zebrafish Show Mild but Significant Changes in Cardiac Anatomy

**Figure 7.**
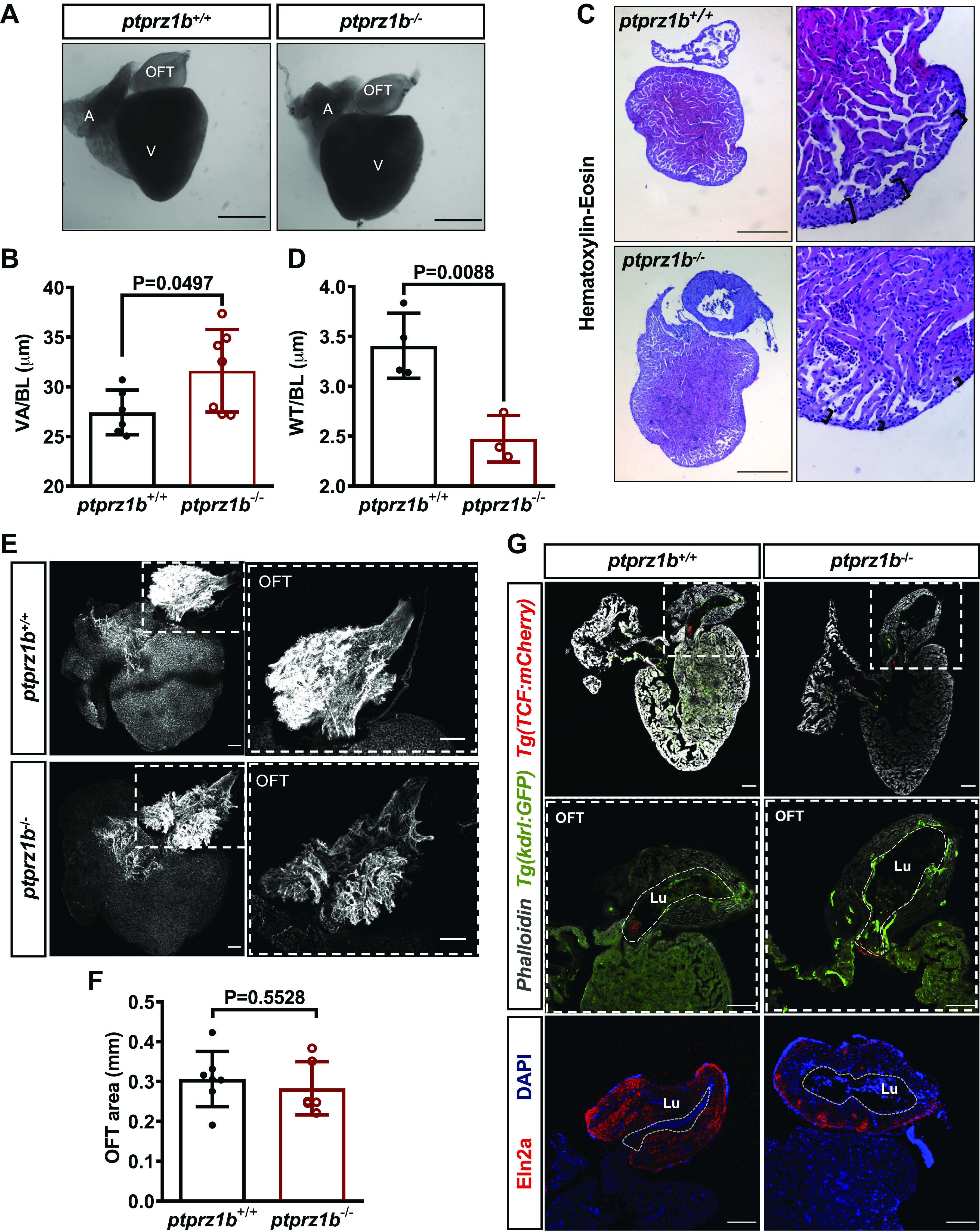
Changes in cardiac anatomy between *ptprz1b^+/+^* and *ptprz1b*^−/−^ adult zebrafish. *A*: representative images of hearts collected from adult *ptprz1b^+/+^* and *ptprz1b*^−/−^ zebrafish. Scale bars correspond to 500 μm. *B*: quantification of ventricular size expressed as means ± SD of ventricular area/body length (VA/BL) (*ptprz1b^+/+^*, *n* = 6 and *ptprz1b*^−/−^, *n* = 7). *C*: representative histology images of adult *ptprz1b*^−/−^ and *ptprz1*^+/+^ hearts after H&E staining. Scale bars correspond to 300 μm. *D*: measurements of wall thickness expressed as means ± SD of wall thickness/body length (WT/BL; *n* = 3). *E*: representative confocal images of whole mounted adult hearts (maximum projections). The OFT structure is highlighted by the dashed white box. Scale bars correspond to 100 μm. *F*: quantification of the OFT area normalized by the total surface area of the respective ventricles and expressed as means ± SD (*ptprz1b^+/+^*, *n* = 7 and *ptprz1b*^−/−^, *n* = 6). *G*: images of adult heart cryosections, in which the OFT’s lumen is marked with white dashed lines. Scale bars correspond to 100 μm. Statistical analysis in all cases was performed by Student’s unpaired *t* test. A, atrium; BA, bulbus arteriosus; H&E, hematoxylin & eosin; Lu, lumen; OFT, outflow track; V, ventricle.

Adult zebrafish heart measurements using whole mount (Fig. 7*A*, quantified in 7*B*) and H&E staining (Fig. 7*C*, quantified in Fig. 7*D*) show that the ventricles of *ptprz1b*^−/−^ zebrafish are enlarged but with thinner compact myocardial layer, pointing out to a cardiac dilation phenotype. Interestingly, whole mounted heart confocal images show that the adult *ptprz1b*^−/−^ zebrafish cardiomyocytes’ size is unaffected (Supplemental Fig. S10) and the cardiac outflow is normal (Fig. 7*E*, quantified in Fig. 7*F*), whereas findings from the Eln2a-labeled cryosections revealed the presence of enlarged lumen within the OFT of mutants (Fig. 7*G*).

### PTPRZ1 Is Expressed in the Human Fetal Heart but Its Expression in the Adult Heart Is Limited

**Figure 8.**
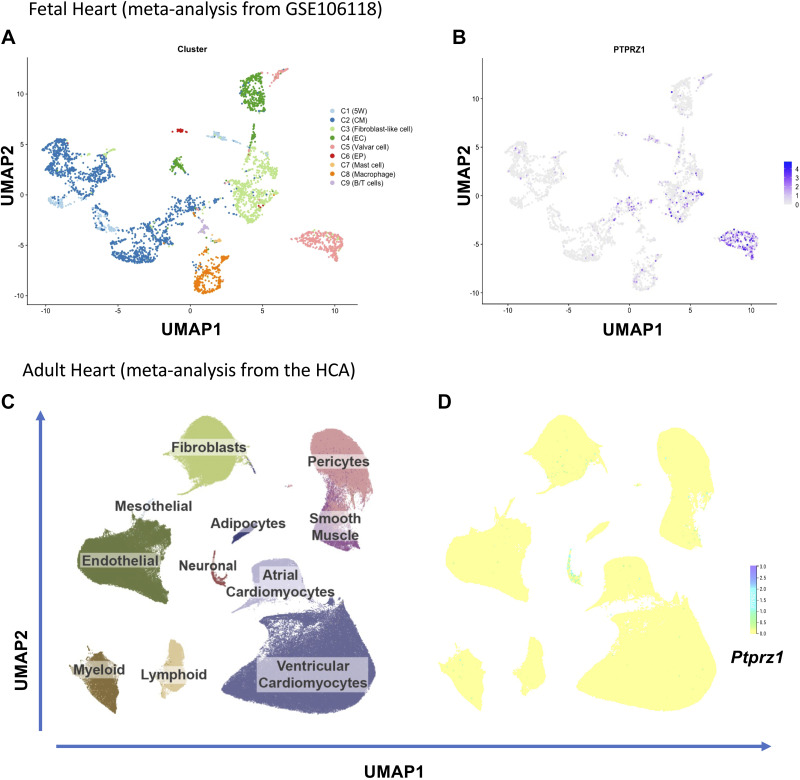
Protein tyrosine phosphatase receptor-**ζ**1 (PTPRZ1) expression in the fetal and adult human heart. *A*: eight clusters (C) of cells as identified in the analysis of single-cell RNASeq of fetal hearts. *B*: uniform manifold approximation and projection (UMAP) representation of *Ptprz1* expression in the developing human heart in different types of cells. *C*: UMAP space colored based on 11 major cardiac cell types across 486,134 cells and nuclei from HCA, as described in https://www.heartcellatlas.org. *D*: UMAP representation of *Ptprz1* expression across 486,134 cells and nuclei from HCA. B/T cells, B and T lymphocytes; CM, cardiomyocytes; EC, endothelial cells; EP, epicardial cells; HCA, Heart Cell Atlas; 5W, 5 weeks hearts.

We assessed the expression levels of PTPRZ1 in the fetal and adult human heart by reanalyzing published single-cell RNAseq data from the study by Cui et al. (33) and the Heart Cell Atlas (HCA) (34). Cui et al. (33) analyzed data from 3,842 cells from different regions and stages of the fetal heart and identified eight clusters of cells (Fig. 8*A*). HCA contains transcriptomes of 486,134 cells and nuclei from 6 different healthy anatomical cardiac regions, including 11 major cardiac cell types (Fig. 8*C*) and 62 different cell states. According to our integration analysis by means of *SCTransform* (35), we observed that during fetal heart development, *Ptprz1* was expressed in a variety of cell types, mainly valvar, fibroblast-like, cardiomyocyte, and endothelial cells (Fig. 8*B*). In the adult heart, however, *Ptprz1* expression is low to negligible in all but neuronal-like cardiac cells (Fig. 8*D*). This observation favors the notion that PTPRZ1 has a role during heart development, whereas it might also control adult cardiac function through its intrinsic innervation, an observation that warrants further investigation.

## DISCUSSION

In the present study, by using two genetically modified animal models, we highlight, for the first time, the role of PTPRZ1 in heart development and function. We did not detect any severe structural defects in mice during development and only mild defects in valve interstitial cell number and outflow tract size in zebrafish. However, cardiac function seems to be predominantly affected in both species. The observation that the orthologous *ptprz1* genes seem to have similar function in both zebrafish and mouse heart implies an evolutionarily conserved, thus potentially important phenotype.

The initial hypothesis that PTPRZ1 may play a role in heart morphogenesis and function came from RNA sequencing data of *Ptprz1*^−/−^ and *Ptprz1*^+/+^ endothelial cells that showed that among the only 26 differentially expressed genes, *Tbx2*, *Tbx20*, *Hand2*, and *Pitx2* are significantly related to heart morphogenesis. Expression of *Tbx2* and *Hand2* is significantly different in *Ptprz1*^−/−^ hearts in a way like that observed in endothelial cells. *Tbx2* acts as a transcriptional repressor of the chamber formation on the sinoatrial border, the AVC, and OFT (1), and heart malformation in a patient has been related to *Tbx2* gene overexpression (36). *Tbx2b* expression is also increased in the *ptprz1*^−/−^ zebrafish heart and may be linked to at least part of the defective cardiac morphogenesis observed in both animal models. *Hand2* expression is critical for the patterning of ventricles (37) and its decreased expression in the *Ptprz1*^−/−^ hearts is also in line with the observed phenotype. It is also in line with the decreased expression of *notch1b* in the *ptprz1*^−/−^ zebrafish hearts, since endocardial HAND2 has been shown to be an integral downstream component of endocardial Notch signaling during cardiogenesis (38). *Tbx20* that acts as transcriptional activator or suppressor of chamber myocardial genes and is essential for heart development, and adult heart integrity, function, and adaptation (1) is not significantly affected in the zebrafish model and is increased in *Ptprz1*^−/−^ mice hearts. It should be noted that a challenge in all the above cases is to study the spatial changes in the expression of the discussed transcription factors in the hearts of *Ptprz1*^−/−^ and *Ptprz1*^+/+^ animal models.

*Ptprz1*^−/−^ mice hearts showed a worse left ventricular systolic function compared with the *Ptprz1*^+/+^ hearts. The total mass of the left ventricle is increased, but the wall thickness is decreased. There is also a decrease in the ratio of the end-systolic to the end-diastolic diameter of the left ventricle, as well as an increase in the ratio of the diameter of the left ventricle to the thickness of its walls. The decreased wall thickness in combination with the decrease *r*/*h* ratio, the increase in both LVESD and LVEDD, and the decrease of the overall LV function do not support the presence of ventricular hypertrophy, in line with the undifferentiated number and size of cardiomyocytes and the absence of signs of fibrosis. The reduced, but within normal values, EF seems inadequate to support a definite diagnosis of dilated cardiomyopathy, since a more reduced EF would be expected. However, the increased ventricular volume of *ptprz1b*^−/−^ zebrafish embryos, together with the ventricular dilation, the thinning of the heart walls in the adult zebrafish, and the lower expression of *Ttn2 (ttna)* that has been associated to dilated cardiomyopathy (39, 40), support the dilated cardiomyopathy hypothesis. In line with this possibility is the description of a precursor phenotype of classical dilated cardiomyopathy with a normal or even marginally reduced EF (41, 42). The decrease in *bmp4* levels and the disrupted specification of *klf2* in *ptprz1b*^−/−^ embryos’ hearts, in combination with the partial inhibition of *wnt* signaling in the AV canal and the low blood flow, revealed a potential problem in the modulation of AV endocardial cushions (43) and the maturation of conduction system (44). Although we did not observe any significant changes in the expression levels of sinoatrial node pacemaker-specific markers *tbx18* and *isl-1* at *ptprz1b*^−/−^ zebrafish embryos using whole embryo RNA, we could not rule out the possibility that there are mild changes of their expression pattern within the heart. Tbx18 expression is significantly decreased in *Ptprz1*^−/−^ compared with *Ptprz1^+/+^* mouse endothelial cells based on our RNAseq data (data not shown). Sinoatrial node function can also occur from dysfunction of other transcription factors, such as *tbx2b*, *bmp4*, or *gata4*, which have been associated with the differentiation of arterial pole cardiomyocytes and the proper function of sinoatrial node and the pacemaker activity AV canal (45, 46) and were shown dysregulated in *ptprz1b*^−/−^ embryos.

Regulation of cardiac-selective transcription factors expression by PTPRZ1 absence favors the notion that PTPRZ1 is primarily important for heart development. How PTPRZ1 affects transcription is not known, but in favor of such notion is the observation that PTPRZ1 has been found in the cell nucleus and nucleoli, interacting with nucleolin (47). Another possibility is that it regulates the tyrosine phosphorylation of numerous substrates in the cell, such as c-Src and Fyn kinases, β-adducin, β-catenin, and protein kinase C delta (PKCδ) (5) that may then affect heart development. Among the PTPRZ1 substrates that have been identified by using a yeast substrate-trapping system is cardiac troponin T (48), which controls the calcium-mediated interaction between actin and myosin and its absence in the developing heart leads to abnormal ventricular morphogenesis (49). It is not known up to date how troponin T phosphorylation affects cardiac morphogenesis, but it has been shown that its phosphorylation at tyrosine 26 accelerates thin filament deactivation and regulates cardiac function, similarly to its serine phosphorylation (50). PTPRZ1 may affect both tyrosine and serine phosphorylation of cardiac troponin T through activation of other kinases, such as PKCδ, as mentioned above.

The notion that PTPRZ1 regulates heart development but not adult heart function is also supported by the observation that the selective PTPRZ1 tyrosine phosphatase inhibitor that we used had no effect on the function of adult hearts but affects the developing heart similarly to PTPRZ1 loss. Altogether, our data suggest that the changes in the heart functions measured in both animal models are most likely due to developmental heart malformation and the *Ptprz1* gene is worthy of screening for congenital heart defects. Further studies to investigate how *ptprz1*^−/−^ hearts respond to stress are warranted and supported by our observation that *Ptprz1*^−/−^ mice are more sensitive to the cardiotoxicity of the anaplastic lymphoma kinase tyrosine kinase inhibitor crizotinib and tolerate half the dose of the drug (22) compared with the suggested dose for mice in the literature (51).

During fetal heart development, besides cardiomyocytes and valvar cells, *Ptprz1* was also expressed in fibroblasts and endothelial cells that are known to be integral components and significant players during cardiogenesis (52, 53). Cardiac fibroblasts derive from both the endocardium and epicardium during embryonic development and are considered to significantly affect myocardial growth, whereas they seem to have little effect in the adult myocardium in health or disease (54). PTPRZ1 has been shown to be highly expressed during embryonic development (5) in line with our data in the present work showing increased expressed in the embryonic heart and is known to affect angiogenesis through downstream activation of numerous signaling molecules (5, 10, 22). The role of PTPRZ1 in fibroblasts functions has not been studied up to date.

In pathologies with enhanced inflammatory signs, such as schizophrenia (55), Parkinson’s disease (56), multiple sclerosis (57), and osteoarthritis (58), there is an increased incidence of cardiovascular events and deterioration of cardiac function. PTPRZ1 has been implicated in the pathophysiology of all the above-mentioned pathologies (59–62), and it would be interesting to study whether the deranged cardiovascular function in such cases is also congenital and relates to differences in the expression of PTPRZ1.

Absence of PTPRZ1 correlates with increased angiogenesis in the mouse heart. This observation is in line with the long-known observation that cardiac capillary density is inversely related to HR (63) and leads to the hypothesis that the increased capillary density without any changes in cardiomyocyte size or heart weight is a secondary effect resulting from the decreased function of the *Ptprz1*^−/−^ heart and the potential resulting hypoxia. Chronic hypoxia has been shown to induce angiogenesis (64) and may also explain the deranged angiogenesis observed in both animal models. However, it cannot be excluded that the observed increased number of capillaries may be due to a direct effect of PTPRZ1 on endothelial cell functions, since increased angiogenesis is also observed in the lungs of *Ptprz1*^−/−^ mice and endothelial cells isolated from *Ptprz1*^−/−^ lungs have enhanced angiogenic activities, such as proliferation, migration, and tube formation in vitro (22).

Taken together, our data highlight PTPRZ1 as a novel regulator of cardiac morphogenesis and subsequent heart function and warrant further studies for the involvement of PTPRZ1 in congenital cardiac pathologies.

## DATA AVAILABILITY

The raw reads of transcriptomic data were deposited in the Gene Expression Omnibus (GEO, accession number: GSE161080) at the National Center for Biotechnology Information (NCBI). On reasonable request, materials can be obtained through an material transfer agreement.

## SUPPLEMENTAL DATA

Supplemental Table S1 and Supplemental Figs. S1–S10: https://doi.org/10.6084/m9.figshare.16924447.v1.

Supplemental Movies S1–S6: http://doi.org/10.6084/m9.figshare.15047994.

## GRANTS

This project was financed by three scholarships (to S.K.-P., P.K., and D.N.) from the Hellenic State Scholarship Foundation (IKY, Operational Program “Human Resources Development-Education and Lifelong Learning,” Partnership Agreement PA 2014-2020, MIS 5000432). This publication has been financed by the Research Committee of the University of Patras.

## DISCLOSURES

No conflicts of interest, financial or otherwise, are declared by the authors.

## AUTHOR CONTRIBUTIONS

D.B. and E. Papadimitriou conceived and designed research; S.K.-P., P.K., D.B., D.N., A.V., S.N., E.A., and C.M.M. performed experiments; S.K.-P., P.K., D.B., D.N., A.V., S.N., E.A., D.B., and E. Papadimitriou analyzed data; S.K.-P., C.H.D., E. Papadaki, G.T., G.H., D.B., and E. Papadimitriou interpreted results of experiments; S.K.-P., P.K., D.B., A.V., E.A., D.B., and E. Papadimitriou prepared figures; S.K.-P., D.B., and E. Papadimitriou drafted manuscript; S.K.-P., C.H.D., G.T., G.H., C.M.M., D.B., and E. Papadimitriou edited and revised manuscript; S.K.-P., P.K., D.B., D.N., A.V., C.H.D., S.N., E. Papadaki, G.T., E.A., G.H., C.M.M., D.B., and E. Papadimitriou approved final version of manuscript. 
